# The Incidence of Hematological Toxicities in Colorectal Cancer Patients Treated With Fluoropyrimidine-Based Regimens at Princess Noorah Oncology Center

**DOI:** 10.7759/cureus.44267

**Published:** 2023-08-28

**Authors:** Mansoor Khan, Sara Alharbi, Shahad Aljuhani, Mariam Tunkar, Amjaad Morya, Abdelmajid Alnatsheh, Majed Alshamrani, Razaz Felemban

**Affiliations:** 1 Pharmaceutical Care Services, Ministry of National Guard Health Affairs, King Abdullah International Medical Research Center, Jeddah, SAU; 2 College of Medicine, King Saud bin Abdulaziz University for Health Sciences, Jeddah, SAU; 3 Pharmacy, Parkview Packnett Family Cancer Institute, Fort Wayne, USA

**Keywords:** colorectal cancer, hematological toxicity, dihydropyrimidine dehydrogenase deficiency, fluoropyrimidines, bolus 5-fu

## Abstract

Background

Fluoropyrimidine-based regimens are used for the management of colorectal cancer, which is the second most common cancer in Saudi Arabia. We aimed to study the incidence of hematological toxicities in colorectal cancer patients treated with fluoropyrimidine and fluoropyrimidine-based regimens at Princess Noorah Oncology Center, King Abdulaziz Medical City- Jeddah, Saudi Arabia.

Methods

A retrospective cohort study that included adult colorectal cancer patients who were treated with fluoropyrimidine-based regimens from January 1, 2018 to December 31, 2018 at Princess Noorah Oncology Center, Jeddah, Saudi Arabia was performed. Our primary objective was to determine the incidence of anemia, neutropenia, and thrombocytopenia in colorectal cancer patients treated with fluoropyrimidines and fluoropyrimidine-based regimens. Secondary objectives were to assess the grade of hematological toxicities associated with 5-fluorouracil (5-FU) use and to determine the frequency of unplanned hospital admissions or emergency department (ED) visits after receiving fluoropyrimidine-based regimens. The collected data contained patients’ characteristics (weight, height, age, gender, and diagnosis), chemotherapy agents, and hematological toxicity-related findings such as absolute neutrophil count, hemoglobin, platelet count, and number of ED visits or hospital admissions during fluoropyrimidine-based chemotherapy regimens.

Results

Of the 570 cycles of the fluoropyrimidine-based regimen received by 68 patients, hematological toxicities were observed in 508 (89.1%) cycles, and grade ≥ 3 grade toxicities were found in 46 (8.1%) cycles. The results demonstrated a statistically significant difference in the incidence of grade 3-4 neutropenia between patients who received bolus administration of 5-FU and those who did not (8.5% vs. 2.3% respectively, p=0.025). The incidence of grade 3-4 anemia was higher in the bolus group (11.3%) compared to the group where bolus was omitted (4.6%); however, the difference was not statistically significant (p=0.059). Furthermore, there was no significant difference among the two groups for grade 3 and grade 4 thrombocytopenia (0.0% with bolus given and 0.7% with bolus omission p=1.00).

Conclusion

Our retrospective study showed that there have been significantly higher grade 3-4 hematological toxicities observed with bolus administration of 5-FU, which confirms the previous reports.

## Introduction

Colorectal cancer is the second most commonly diagnosed cancer in Saudi nationals, with an incidence of 1908 cases in 2018 representing about 12.2% of all cancers in the country and the most common among males as per the Saudi cancer incidence report of 2018 [1]. However, the most recent cancer incidence in the country by the Global Cancer Observatory Report 2020 has shown that colorectal cancer is the most diagnosed cancer with an incidence of 4007 cases representing 14.4% of the total number of cancer cases, with an 8.3% and 6.7% mortality rate for colon cancer and rectum cancer respectively, placing it as the third and fourth leading cause of cancer-related deaths in Saudi Arabia [2].

Globally, colorectal cancer is the third most diagnosed cancer in males and females, and the second leading cause of cancer-related deaths [3]. Fluoropyrimidines are a class of anti-cancer drugs that include 5-fluorouracil (5-FU) and its prodrug capecitabine. Fluoropyrimidine-based chemotherapy regimens are the standard of care treatment for colorectal cancer patients in adjuvant and advanced settings. According to the Saudi gastrointestinal cancer clinical guidelines, 5-FU or capecitabine is included in the treatment plan of each type of gastrointestinal (GI) cancer at different stages of the disease as a stand-alone drug or as a part of a regimen. 5-FU is commonly administered as a bolus injection followed by continuous infusion in certain regimens used for the management of colorectal cancer [4].

The fluoropyrimidines are broken down into three metabolites, fluorodeoxyuridine monophosphate (FdUMP), fluorodeoxyuridine triphosphate (FdUTP), and fluorouridine triphosphate (FUTP) that act through these mechanisms [5,6]. Capecitabine is a prodrug of fluorouracil. It undergoes hydrolysis in the liver and tissues to form fluorouracil which is the active moiety. Fluorouracil is a pyrimidine analogue antimetabolite that interferes with DNA and RNA synthesis; after activation, F-UMP (an active metabolite) is incorporated into RNA to replace uracil and inhibit cell growth; the active metabolite F-dUMP, inhibits thymidylate synthetase, depleting thymidine triphosphate (a necessary component of DNA synthesis). Fluorouracil appears to be phase-specific for the G and S phases of the cell cycle [7]. This interference with the DNA replication process leads to damage to the cell’s DNA and will eventually result in cell death. FdUTP acts by incorporating into the DNA as a pyrimidine analogue, replacing thymine and leading to DNA damage. FUTP acts by incorporating into the RNA by replacing uracil and leading to RNA damage. The pharmacodynamic pathway of 5-FU differs based on the dosage schedule, bolus acts by incorporating it into RNA, whereas continuous infusion acts mainly on the inhibition of thymidylate synthase and incorporating it into the DNA [5,6].

Dihydropyrimidine dehydrogenase (DPD) enzyme is the rate-limiting step of fluoropyrimidine catabolism. Nearly, 80%-85% of 5-FU is catabolized by DPD in the liver while 5-20% is excreted intact in the urine [7]. Only 1-3% of 5-FU enters the cytotoxic pathway. DPD converts 5-FU to dihydrofluorouracil (DHFU). DHFU is then converted to fluoro-β-ureidopropionate (FUPA) by dihydropyrimidinease (DPYS). Subsequently, FUPA is converted to fluoro-β-alanine (FBAL) by β-ureidopropionase (UPB1). Deficient activity in these enzymes can result in severe 5-FU toxicity. In fact, the FDA-approved drug label for 5-FU states that life-threatening toxicities are highly associated with DPD deficiency [8]. In a recent case report, a Saudi patient with rectal adenocarcinoma who experienced severe toxicities secondary to a standard dose of 5-FU-based chemotherapy was found to have a DPYD polymorphism [9].

Fluoropyrimidine-based regimens have a high risk of causing severe toxicity in up to 40% of the patients, leading to the discontinuation of potentially effective anticancer therapy, and often requiring hospitalization [10]. To our knowledge, the incidence of toxicities related to 5-FU in the Saudi population has not yet been reported. Toxicities reported in the literature include mucositis, diarrhea, leukopenia, neutropenia, hand-foot syndrome, and cardiotoxicities [11-14]. A study published in 2012 evaluating hematological toxicities reported that 70% of patients presented with grade 1 or higher neutropenia and/or leukopenia. Grade 3-4 hematological toxicities were observed in 63% of patients, and 52% presented as neutropenia, 12% as leukopenia, and 2 % as thrombocytopenia [15]. There is also variation in the type of toxicity exhibited by patients treated with different dose schedules, bolus 5-FU was more associated with neutropenia, whereas continuous infusion 5-FU was more associated with gastrointestinal toxicity, skin toxicity, and hand-foot syndrome [16].

Princess Noorah Oncology Center (PNOC) is one of the biggest tertiary care referral oncology centers in the western region of Saudi Arabia where all kinds of cancer diseases including colorectal cancers are treated with fluoropyrimidine-based regimens. It was hypothesized that 5-FU causes hematological toxicity in a number of cancer patients at PNOC and the practice changed toward dropping 5-FU bolus for the majority of patients in the palliative treatment setting. Therefore, this study retrospectively determines the incidence of hematological toxicities specifically anemia, neutropenia, and thrombocytopenia in colorectal cancer patients who were exposed to fluoropyrimidine-based regimens during the period January 1, 2018 through December 31, 2018 at PNOC, King Abdulaziz Medical City, Jeddah.

## Materials and methods

This retrospective cohort study was done at PNOC, King Abdulaziz Medical City, Jeddah, Saudi Arabia. The study was approved by the ethical committee and Institutional Review Board (IRB) with IRB approval number SP20/051/J. It included all male and female colorectal cancer patients who were 18 years and older and had received six or more cycles of either 5-FU or capecitabine-based regimens between January 2018 and December 2018. All pregnant women were excluded from the study. 

Our primary objective was to determine the incidence of anemia, neutropenia, and thrombocytopenia in colorectal cancer patients treated with fluoropyrimidines and fluoropyrimidine-based regimens. One of the key secondary endpoints was to assess and compare the grade of hematological toxicities associated with 5-FU use among colorectal cancer patients when 5-FU bolus was given versus when omitted. Other secondary endpoints included determination of the frequency of unplanned hospital admissions or emergency department (ED) visits after receiving fluoropyrimidine-based regimens. 

The data of all diagnosed colorectal cancer patients treated at PNOC in King Abdulaziz Medical City registered between 1 January 2018 and 31 December 2018 who received fluoropyrimidine-based regimens were collected from the hospital information system (BESTCare and ARIA) using a data collection sheet. The collected quantitative variables were age, weight in Kg, height in cm, percentage of dose reduction if any, complete blood counts with differentials including white blood cell count, absolute neutrophil count, hemoglobin, platelet, and the number of ED visits or hospital admissions after receiving fluoropyrimidine-based regimens. Qualitative variables included gender, cancer type, cancer stage, and outcome of toxicity. Categorical variables were presented as percentages, while parametric approaches were used to describe the numerical variables. 

Grading of hematological toxicities such as neutropenia, anemia, and thrombocytopenia following each cycle were assessed, using the National Cancer Institute Common Terminology Criteria for Adverse Events (CTCAE) version 5 of World Health Organization (WHO) and National Cancer Institute (NCI). 

The Fisher exact test was used to compare hematological toxicities resulting from cycles where bolus was given versus when bolus was omitted. A p-value less than 0.05 was considered statistically significant. The data were analyzed using IBM SPSS Statistics for Windows, Version 20 (Released 2011; IBM Corp., Armonk, New York, United States).

## Results

In our retrospective study, we reviewed 570 cycles of the 5-FU-based regimen received by 68 patients with a mean age of 58 (SD±12) years and a female/male ratio of 39/29 (57.4% vs 42.6%, Table 1). A total of 518 cycles were 5-FU-based regimens and 52 cycles were capecitabine-based regimens. All 570 cycles were evaluated for the primary objective. Hematological toxicities were observed in 508 (89.1%) cycles and grade 3-4 toxicity was found in 46 (8.1%) cycles. Different hematological toxicities including anemia, thrombocytopenia, and neutropenia observed with the 5-FU-based regimen were recorded and analyzed by grades (Table 2). Anemia was overall the most common hematological toxicity observed in 441 (77.4%) cycles and the most common grade 3-4 toxicity observed in 29 (5.0%) cycles of 5-FU-based regimes. Thrombocytopenia was observed in 85 cycles (14.7%) with grade 3-4 thrombocytopenia observed only in three cycles (0.5%). All-grade neutropenia was seen in 177 patients (31.1%) with grade 3-4 neutropenia developed in 17 cycles (3%).

**Table 1:** Patients' demographics CAPOX: Capecitabine and oxaliplatin; CAPIRI: capecitabine and irinotecan; FOLFIRI: folinic acid (leucovorin), fluorouracil (5-FU), and irinotecan; FOLFIRINOX: folinic acid (leucovorin), fluorouracil (5-FU), irinotecan, and oxaliplatin; FOLFOX: folinic acid (leucovorin), fluorouracil (5-FU), and oxaliplatin; FOLFOXIRI: folinic acid (leucovorin), fluorouracil (5-FU), oxaliplatin, and irinotecan; Mod-FOLFOXIRI: modified FOLFOXIRI

Demographic variables	Frequency	Percentage
Age (Y) (mean ± SD)	(58±12)	
Weight (Kg) (mean ± SD)	(67 ± 19.8)	
Height (cm) (mean ± SD)	(159.75 ± 8.662)	
Gender		
Male	29	42.6%
Female	39	57.4%
Diagnosis		
Colon	51	75.0%
Rectal	15	22.1%
Mixed	2	2.9%
Stage		
Stage I	0	0.0%
Stage II	2	2.9%
Stage III	6	8.8%
Stage IV	60	88.2%
Chemotherapy Regimen		
FOLFIRI	21	3.7%
FOLFIRI-Bevacizumab	118	20.7%
FOLFIRI-Cetuximab	98	17.2%
FOLFIRI-Panitumumab	16	2.8%
FOLFOX	38	6.7%
FOLFOX- Bevacizumab	115	20.2%
FOLFOX- Cetuximab	26	4.6%
FOLFOX- Panitumumab	56	9.8%
FOLFOXIRI- Panitumumab	8	1.4%
FOLFIRINOX	8	1.4%
Mod-FOLFOXIRI- Bevacizumab	14	2.5%
CAPIRI- Bevacizumab	8	1.4%
CAPOX	32	5.6%
Capecitabine / Bevacizumab	12	2.1%
Bolus Administration		
Given	71	14.1%
Omitted	433	85.9%

**Table 2:** Types and grades of different hematological toxicities observed

Type of hematological toxicity (N= 570 cycles)	Grade 1 N (%)	Grade 2 N (%)	Grade 3 N (%)	Grade 4 N (%)	All grades N (%)
All hematological toxicities			46(8.1)		508(89.1)
Anemia	280 (49.1)	132 (23.2)	27 (4.7)	2 (0.3)	441 (77.4)
Thrombocytopenia	75 (13.2)	7 (1.2)	3 (0.5)	0 (0.0)	85 (14.7)
Neutropenia	99 (17.4)	61 (10.7)	13 (2.3)	4 (0.7)	177 (31.1)

We also evaluated the administration pattern of 5-FU regarding its bolus administration to see whether 5-FU bolus was given or omitted in all cycles. 5-FU bolus was given in 71 cycles and it was omitted in 433 cycles, whereas 5-FU bolus was not applicable in 66 cycles as it was not part of the regimens in these 66 cycles. Then we looked at the difference in the incidence of hematological toxicities among the cycles where 5-FU bolus was received vs bolus was omitted. The results indicated that there was a statistically significant difference in the frequency of grade 3- 4 neutropenia among the two groups (grade 3-4 neutropenia was 8.5% with 5-FU bolus given and 2.3% with 5-FU bolus omitted; p= 0.025. The incidence of grade 3-4 anemia was higher in the bolus group (11.3%) compared to the group where bolus was omitted (4.6%); however, the difference was not statistically significant (p=0.059), while there was no significant difference among the two groups for grade 3 and grade 4 thrombocytopenia (0.0% with bolus given and 0.7% with bolus omission p=1.00). ED visits or hospital admissions among patients receiving 5-FU-based regimens were recorded. We found that 18 (26.4%) cycles where 5-FU bolus was given and 8 (11.7%) cycles where 5-FU bolus was omitted had documented ED visits and hospital admissions of the patients (Table 3).

**Table 3:** 5-FU bolus given vs omitted 5-FU: 5-Fluorouracil

Bolus given or omitted (N= 504 cycles)	Number of cycles	Grade 3-4 neutropenia N (%)		Grade 3-4 anemia N (%)	Grade 3-4 thrombocytopenia N (%)
Bolus given	71	6 (8.5)	8 (11.3)		0 (0.0)
Bolus omitted	433	10 (2.3)	20 (4.6)		3 (0.7)
p-value		0.025	0.059		1.00

## Discussion

A systematic review has reported that patients with the A allele had an approximately 2.4- and 1.9-fold higher chance of hematological toxicity and neutropenia in comparison to those with the GG genotype. Hematological toxicity is one of the reasons for chemotherapy to be stopped while severe neutropenia is a deadly outcome associated with chemotherapy. These toxicities can increase mortality and morbidity and mandate dose reduction and treatment interruption which may compromise the clinical outcomes. A systematic review and meta-analysis provided updated data regarding fluoropyrimidine-associated toxicity dangers in patients with the A allele of rs1801160 with greater chances of clinical modifications based on genotypic profiles (DPYD Genetic Polymorphism) for fluoropyrimidine therapy [17].

The significance of hematological toxicities was acknowledged in a study published in France. Approximately, 497 solid-tumor patients receiving 5-FU were observed for serious adverse events. The serious adverse event incidence rate was 19.3% (95%CI 16-23%) including one toxic death (0.2%, 95%CI 0-1%) during the first two cycles. The rate increased over the first six months of treatment to 32.2% (95%CI 28-36%). Among these adverse events, 53.4% were hematologic toxicities [18].

Our study confirmed the existence and significance of hematological toxicities among colorectal cancer patients, who received 5-FU and 5-FU-based regimens, in Princess Noorah Oncology Center at King Abdulaziz Medical City- Jeddah (2018-2019). The study has shown that 5-FU bolus was given in 71 cycles and was omitted in 433 cycles, whereas 5-FU bolus was not applicable in 66 cycles as it was not part of the regimens such as Modified FOLFIXIRI (14 cycles), CAPIRI-bevacizumab (eight cycles), CAPOX (32 cycles), and capecitabine -bevacizumab (12 cycles) as shown in Table 1. The majority of the patients who received 5-FU bolus have developed more hematological toxicities, than the patients whose bolus was omitted. 5-FU bolus was given in 71 cycles of 5-FU-based regimens and 66 cycles out of these 71 cycles (92%) resulted in hematological toxicities.

We also found that patients who received cycles of 5-FU may develop hematological toxicity of any grade, commonly; anemia 441 (77.4%) and neutropenia 177 (31.1%). The most common finding of all grades of hematological toxicity was anemia observed in 441 (77.4%) cycles of 5-FU. While neutropenia was found in 177 (31.1%) cycles of 5-FU, thrombocytopenia was seen in 85 (14.7%) cycles of 5-FU. Grade 3-4 anemia, neutropenia, and thrombocytopenia were found in 5%, 3%, and 0.5% cycles, respectively. There were significantly more grade 3-4 anemia and neutropenia among the cycles where bolus of 5-FU was given in comparison to the cycles where bolus was omitted which confirms the previous reports on the toxicity patterns of bolus administration of 5-FU.

Nowadays, the FDA approved an important assay test to screen cancer patients for DPD deficiency before they receive 5-FU. The DPD enzyme is critical for the metabolism of fluoropyrimidine drugs. With deficient enzyme function, patients can experience severe toxicities with standard doses of fluoropyrimidine chemotherapy. While the incidence of DPD deficiency is relatively low, ranging from 1 to 7 percent of the population depending on ancestry, the consequences are potentially fatal [19].

Another tool for DPD deficiency screening uses uracil doses and close cutoff measures. The tool is directed to patients under adjuvant CRC treatment [20]. Many genetic forms of DPD result in different 5-FU plasma levels of toxicity and cancer persistence. For example, combining Gimeracil and Eniluracil with 5-FU improves treatment outcomes and prevents cancer resistance. Some of these combinations are currently approved for use and others are still going through multiple trials to make sure 5-FU gets high bioavailability [21].

The use of chemotherapy may stop the growth of cancer cells in addition to normal cells that are characterized by a high division rate. This leads to defects in many fast-producing sites in the body. One crucial example chemotherapy may affect is the bone marrow which leads to defects in clotting pathways, immunity system, hemoglobin level disturbance, and neutropenia with other hematological toxicities [22].

Our results are limited by the absence of laboratory screening for DPD deficiency in the sampled patients to confirm the cause of the toxicity and distinguish DPD-deficient patients from others who have different etiologies.

Our study provides a significant finding and confirms the results of 5-FU hematological toxicities. Our finding suggests omitting the 5-FU bolus in our cancer patients as its use has been associated with a significant increase in grade 3-4 hematological toxicities such as neutropenia and anemia. At the same time, recently published data suggested that 5-FU bolus dosing was not associated with an overall survival benefit among patients with metastatic colorectal cancer. However, future studies are required to determine the role of bolus dosing in the adjuvant setting and its impact on other clinically relevant outcomes [23]. We suggest having a DPD screening assay in our cancer patients requiring fluoropyrimidine-based regimens as some of our patients who had grade 3-4 hematological toxicities might have had DPD deficiency. We need to screen our colorectal cancer patients to avoid these complications and improve their quality of life.

The fact that this study was conducted retrospectively and at a single center limits the generalizability of the findings to other populations and settings. Therefore, the results should be interpreted with caution and further research is needed to confirm the findings in larger, prospective studies conducted across multiple centers.

## Conclusions

Our study revealed compelling evidence of significant hematological toxicities, with anemia emerging as the predominant toxicity observed among cancer patients who received 5-FU. The bolus administration of 5-FU showed a significant role in increasing the risk of hematological toxicities. We recommend future prospective studies to confirm the effect of omitting bolus 5-FU on the clinical outcomes in colorectal cancer patients in adjuvant and metastatic settings.
